# Exploring Differentially Expressed Genes by RNA-Seq in Cashmere Goat (*Capra hircus*) Skin during Hair Follicle Development and Cycling

**DOI:** 10.1371/journal.pone.0062704

**Published:** 2013-04-30

**Authors:** Rongqing Geng, Chao Yuan, Yulin Chen

**Affiliations:** 1 College of Animal Science and Technology, Northwest A&F University, Yangling, People’s Republic of China; 2 College of Life Science and Technology, Yancheng Teachers University, Yancheng, People’s Republic of China; University of North Carolina at Charlotte, United States of America

## Abstract

Cashmere goat (*Capra hircus*) hair follicle development and cycling can be divided into three stages: anagen, catagen and telogen. To elucidate the genes involved in hair follicle development and cycling in cashmere goats, transcriptome profiling of skin was carried out by analysing samples from three hair follicle developmental stages using RNA-Seq. The RNA-Seq analysis generated 8487344, 8142514 and 7345335 clean reads in anagen, catagen and telogen stages, respectively, which provided abundant data for further analysis. A total of 1332 differentially expressed genes (DEGs) were identified, providing evidence that the development of hair follicles among the three distinct stages changed considerably. A total of 683 genes with significant differential expression were detected between anagen and catagen, 530 DEGs were identified between anagen and telogen, and 119 DEGs were identified between catagen and telogen. A large number of DEGs were predominantly related to cellular process, cell & cell part, binding, biological regulation and metabolic process among the different stages of hair follicle development. In addition, the Wnt, Shh, TGF-β and Notch signaling pathways may be involved in hair follicle development and the identified DEGs may play important roles in these signaling pathways. These results will expand our understanding of the complex molecular mechanisms of hair follicle development and cycling in cashmere goats and provide a foundation for future studies.

## Introduction

Cashmere goats are only found in specific areas in the world, distributed mainly in China, Mongolia, Afghanistan and Iran. Cashmere is the term for the soft down undercoat of cashmere goats. The fleece of cashmere goats is made up of two distinct fibres: the coarse outer hair and the fine cashmere undercoat. The coarse outer hair is called guard hair. The fine undercoat is the source of cashmere fibres for clothing, which is called down. The down is produced by secondary follicles, the guard hair by the primary follicles [1]–[3].

The post-natal hair follicle of mammalian species undergoes a cycling of growth (anagen), regression (catagen) and rest (telogen) [4]–[6]. Researches on the molecular mechanisms that control hair follicle cycling, especially the relevant signaling pathways, have advanced in the recent ten years [7]–[11]. It is now widely accepted that hair follicle transformation during cycling is caused by alterations in the local signaling milieu. There are key regulators that build up local gradients with competing stimulating and inhibitory signals. Rhythmic changes of signal transducers in the key compartments of the follicle are thought to drive cyclic hair follicle transformation. The secondary follicle cycling of cashmere goat is also composed of anagen, catagen and telogen. However, reports on the molecular mechanisms regulating the secondary follicle cycling of cashmere goats are rare in the corresponding field of study.

RNA-Seq technology is a high-throughput sequencing platform allowing us to detect transcripts with low abundance, identify novel transcript units, and reveal their differential expression between different samples [12]–[14]. To date, RNA-Seq technology has not been used to analyse hair follicle cycling in cashmere goats.

China has a centuries-old history of breeding cashmere goats and owns abundant cashmere goat breeding resources, and it is the largest cashmere-producing country. This study is a genome-wide expression analysis using RNA-Seq to explore DEGs related to cashmere goat hair follicle development and cycling in a cost-effective manner. It includes identifying genes expressed in a stage-specific manner, defining clusters of genes showing similar patterns of temporal expression, and identifying stage-specific candidate genes for additional functional analysis. The genes of the different expression clusters associated with different functional categories clearly indicate the molecular and cellular events involved in hair follicle development and cycling.

## Materials and Methods

### Experimental Animals and Sample Collection

The Shaanbei White cashmere goat is a new outstanding breed for its excellent cashmere production performance. Experimental cashmere goats were obtained from the Shaanbei Cashmere Goats Engineering Technology Research Center of Shaanxi Province, China. All cashmere goats were raised by feeding practices according to the cashmere goat standard. Ten adult individuals (five males and five females, two years old) were randomly selected, and any two or more individuals with a traceable phylogenetic relationship were avoided in the sampling process. Skin samples were collected from the right mid-side of each sampled goat at three hair follicle developmental stages (anagen, catagen and telogen). The same goats were used for each developmental stage. The skin tissues were rinsed in ice-cold DEPC-treated water and were cut into small pieces, and then submerged in RNAlater (ABI, USA) and frozen at −70°C until further processing. All experimental procedures with goats used in the present study had been given prior approval by the Experimental Animal Manage Committee of Northwest A&F University under contract (2011-31101684).

### RNA Extraction and Quality Analysis

Total RNA was extracted from the collected skin tissues using Trizol reagent (Invitrogen, USA) following the manufacturer’s instructions after grinding them under liquid nitrogen. The RNA concentration and quality were further determined using the Agilent 2100 bioanalyzer. The extracted total RNA was stored at −70°C for later use.

### Library Preparation and Sequencing

The total RNA of all skin samples at each developmental stage was pooled prior to library preparation in each experimental group. Equimolar quantities of RNA from each skin sample were combined into one pool. mRNA selection, library preparation and sequencing were performed at BGI-Shenzhen on an Illumina HiSeq™ 2000 sequencer according to the manufacturer’s specifications. Briefly, mRNA was enriched by using the oligo (dT) magnetic beads after extracting the total RNA from the samples. Adding the fragmentation buffer, the mRNA was interrupted to short fragments (about 200 bp), then the first strand cDNA was synthesised by random hexamer-primer using the mRNA fragments as templates. Buffer, dNTPs, RNase H and DNA polymerase I were added to synthesise the second strand. The double-stranded cDNA was purified with the QiaQuick PCR extraction kit and washed with EB buffer for end repair and single nucleotide A (adenine) addition. Finally, sequencing adaptors were ligated to the fragments. The required fragments was purified by agarose gel electrophoresis and enriched by PCR amplification. The library products were used for the final sequencing reaction.

### Mapping Reads to the Reference Genome

Two databases were employed for sequence analysis, the ensemble cattle (*Bos taurus*) reference gene (ftp://hgdownload.cse.ucsc.edu/goldenPath/bosTau6/database/refGene.txt.gz) and reference genomic DNA (ftp://hgdownload.cse.ucsc.edu/goldenPath/bosTau6/bigZips/bosTau6.fa.gz).

Sequencing-received raw image data were transformed by base culling into sequence data. Prior to mapping reads to the reference database, we filtered all sequences to remove adaptor sequences and low-quality sequences (the percentage of low quality bases with a quality value ≤5 was >50% in a read). The remaining reads were aligned to the cattle genome using SOAPaligner/soap2 [15], allowing up to two base mismatches.

### Normalized Expression Levels of Genes and Screening of DEGs

The mapped read counts for each gene were normalized for RNA length and for the total read number in the lane according to reads per kilobase of exon model per million mapped reads (RPKM), which facilitates the comparison of transcript levels between samples [16]. The cutoff value for determining gene transcriptional activity was determined based on a 95% confidence interval for all RPKM values for each gene.

A rigorous algorithm has been developed to identify differentially expressed genes between two samples by BGI based on ‘The significance of digital gene expression profiles’ [17]. We used a *P*-value corresponding to a differential gene expression test at statistically significant levels [18]. “FDR (False Discovery Rate) ≤0.001 and the absolute value of log2Ratio ≥1” were used to identify DEGs as the threshold.

### Expression Pattern, Gene Ontology (GO) and Pathway Enrichment Analysis of DEGs

Cluster analysis of gene expression patterns was performed with cluster software [19] and Java Treeview software [20]. GO functional enrichment analysis was carried out using Blast2GO (version 2.3.5) (http://www.blast2go.org/). KEGG pathway analyses were performed using Cytoscape software (version 2.6.2) (http://www.cytoscape.org/) with the ClueGO plugin (http://www.ici.upmc.fr/cluego/cluegoDownload.shtml) [21].

### Validation of RNA-Seq Data

To confirm the differential expression of genes revealed by RNA-Seq, six genes identified to be expressed differentially among different development stages were chosen randomly for qPCR validation. The primers designed for qPCR analysis were listed in Table 1 and β-actin was used as a reference control.

**Table 1:** List of primers used in qPCR analysis.

GeneID	Gene	Forward primer (5′–3′)	Reverse primer (5′–3′)
NM_174044	DSC1	GTCCACCAGCCTAATCCAGT	CCCTGACTGTCTGAGAGCAA
NM_001076936	ACADL	CTGGAAGTGATTTGCAAGGA	TGAGCCTCTCGATTTGTGAC
NM_001083486	CPZ	GATCTGGCAACACAACAAGG	ATGCCCTTGACTAAGATCCG
NM_001076027	HSPB6	GCTGCTGGATGTGAAACACT	CAATGTATCCGTGCTCATCC
NM_001045875	EGR1	AGCAGCCTTCACTCACTCCT	TGGGTTTGATGAGCTGAGAC
NM_001046193	ATF3	CAGAGTGCCTGCAGAAAGAG	TTCTGAGCCCGAACAATACA
NM_173979	β-actin	TGAACCCCAAAGCCAACC	AGAGGCGTACAGGGACAGCA

The qPCR was carried out on an iQ5 system (Biorad, USA) using SYBR Premix Ex Taq (TaKaRa, China) according the manufacturer’s instructions. The thermal cycling conditions used in the qPCR were 95°C for 3 min, followed by 40 cycling of 95°C for 5 s and 60°C for 1 min. The specificity of the SYBR green PCR signal was confirmed by melting curve analysis. There were three biological and technical replicates, respectively. Relative quantification analyses were performed using the comparative CT method, and the relative gene expression was calculated by using the 2^−ΔΔCt^ method [22].

## Results

### Massively Sequencing and Aligning to the Reference Genome

**Figure 1:**
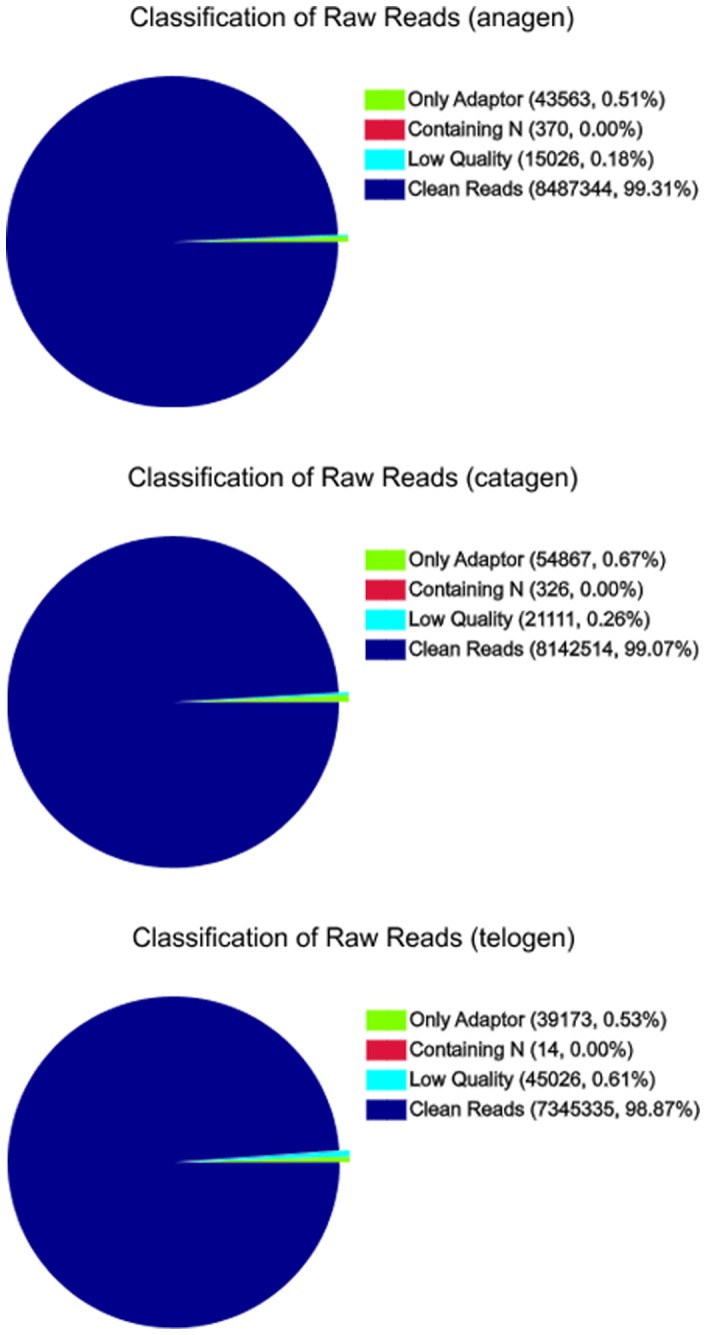
Classification of total raw reads at different developmental stages. After filtering the only adaptor sequences, containing N sequences and low quality sequences, the three RNA-Seq libraries still generated over 7.3 million clean reads in each library, and the percentage of clean reads among raw tags in each library ranged from 98.87% to 99.31%.

To maximise the coverage of cashmere goat skin mRNA by RNA sequencing, RNA libraries were constructed by pooling RNA isolated from different individuals as a sample library at different hair follicle developmental stages. These RNA-Seq libraries generated over 7.4 million raw reads from each library. After filtering the only adaptor sequences, those containing N sequences and low quality sequences, the three RNA-Seq libraries still generated over 7.3 million clean reads in each library, and the percentage of clean reads among raw tags in each library ranged from 98.87% to 99.31% (Fig. 1).

Each stage was represented by approximately seven million reads, a tag density sufficient for the quantitative analysis of gene expression. Of the total reads, more than 57% matched either to a unique or to multiple genomic locations of each stage; the remaining were unmatched (Table 2), because only reads aligning entirely inside exonic regions will be matched (reads from exon-exon junction regions will not be matched).

**Table 2:** Summary of read numbers based on the RNA-Seq data from cashmere goat hair follicle development and cycling.

	Anagen	Catagen	Telogen
Total reads	8487344	8142514	7345335
Mapped reads	5058671 (59.60%)	4946668 (60.75%)	4220093 (57.45%)
Unique match	4236004 (49.91%)	4141919 (50.87%)	3483757 (47.43%)
Multi-position match	822667 (9.69%)	804749 (9.88%)	736336 (10.02%)
Unmapped reads	3428673 (40.40%)	3195846 (39.25%)	3125242 (42.55%)

### Analysis of DEGs at the Different Developmental Stages

**Figure 2:**
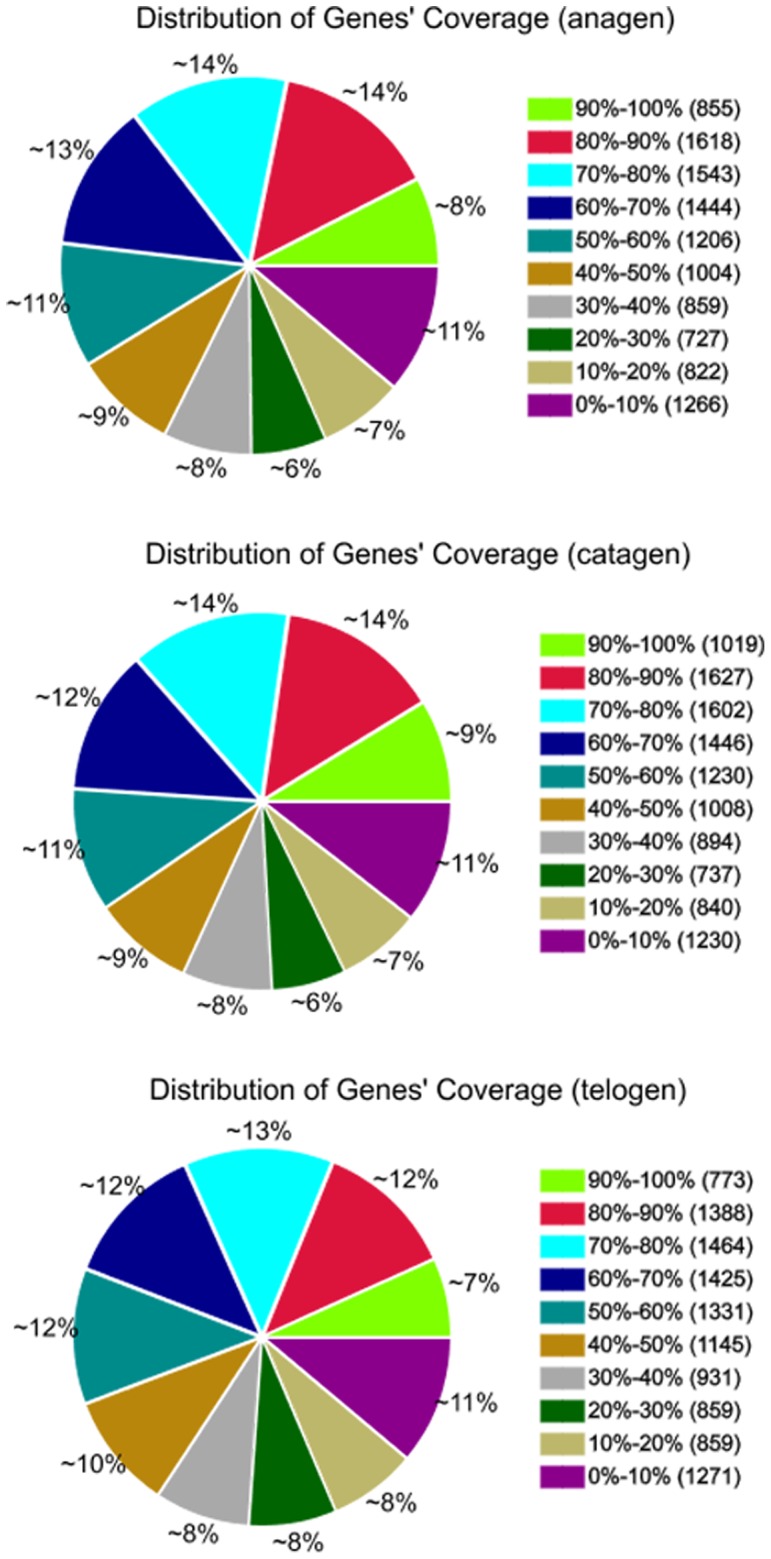
Percent of coverage representing the percentage of genes expressed at each of the stages. The distribution of distinct reads over different read abundance categories showed similar patterns for all the three RNA-Seq libraries. The similarity distribution had a comparable pattern with about 20% of the sequences having an 80% similarity, while approximately 80% of the hits had a similar range.

Gene coverage is the percentage of a gene covered by reads. This value is equal to the ratio of the base number in a gene covered by unique mapping reads to the total bases number of that gene. As shown in Figure 2, the distribution of distinct reads over different read abundance categories showed similar patterns for all the three RNA-Seq libraries. The similarity distribution had a comparable pattern with approximately 20% of the sequences having a similarity of 80%, while approximately 80% of the hits had a similar range.

**Figure 3:**
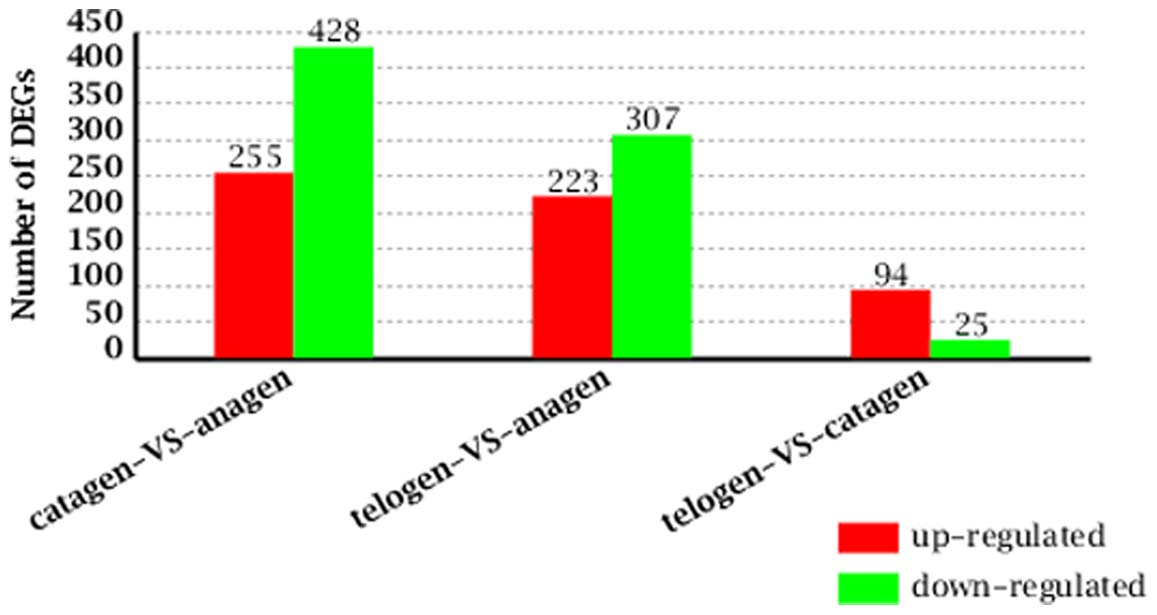
The numbers of DEGs between different developmental stages. Between the anagen and catagen libraries, there were 255 upregulated genes and 428 downregulated genes; Between the anagen and telogen libraries, there were 223 upregulated genes and 307 downregulated genes, while there were 94 upregulated genes and 25 downregulated genes between the catagen and telogen libraries.

To identify the DEGs of hair follicle development and cycling at different stages, the differences in gene expression patterns were analysed for the pairs of anagen and catagen, anagen and telogen, catagen and telogen. A total of 683 genes were differentially expressed between the anagen and catagen libraries (Fig. 3 and Table S1), with 255 upregulated and 428 genes downregulated. Between the anagen and telogen libraries, 530 genes were differentially expressed, including 223 upregulated and 307 downregulated genes (Fig. 3 and Table S2). When we compared the catagen and telogen libraries, 119 DEGs were found, with 94 upregulated and 25 downregulated (Fig. 3 and Table S3). This suggests that the differentiation of expressed genes between anagen and catagen is larger than that between anagen and telogen, while a relatively smaller differentiation arises between catagen and telogen.

### Clustering of DEGs at the Three Developmental Stages

**Figure 4:**
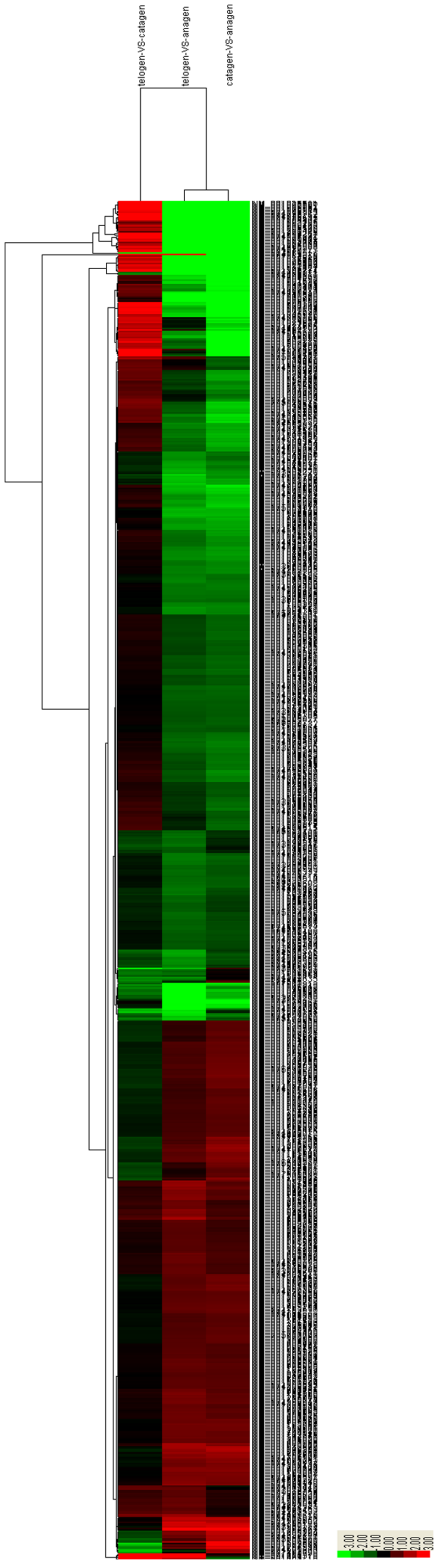
Hierarchical cluster analysis of gene expression based on log ratio RPKM data. The red color represents upregulated genes and the green color represents downregulated genes. There were clusters with relatively minor differences between anagen vs. catagen and anagen vs. telogen, but marked differences with catagen vs. telogen.

Hierarchical clustering was performed to group genes according to similarity in pattern of gene expression [23]. Two-dimensional hierarchical clustering classified 1332 DEGs into several expression cluster groups (Fig. 4) according to the similarity of their expression patterns, representing the number of profiles indicated using figure of merit analysis. Visual inspection of these expression groups suggested diverse and complex patterns of regulation. These clusters contained genes positively or negatively modulated throughout the whole time course, while genes expressed at the three stages of hair follicle development showed certain differences. There were clusters with relatively minor differences between anagen vs. catagen and anagen vs. telogen, but marked differences with catagen vs. telogen.

### Functional Classification Analysis

**Figure 5:**
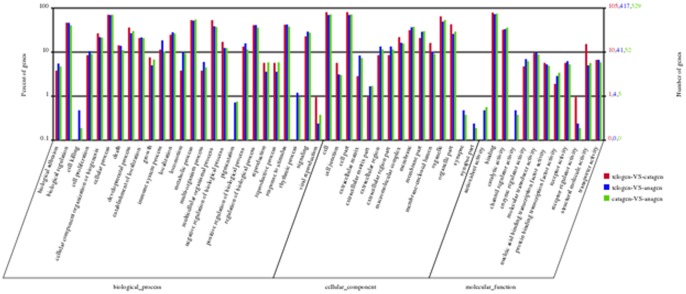
GO functional analysis of DEGs based on RNA-Seq data. The results were summarised in three main categories: biological process, cellular component and molecular function. Among these groups, the terms cellular process, cell & cell part and binding were dominant in each of the three main categories, respectively.

Based on sequence homology, 1332 DEGs could be categorised into 53 functional groups (Fig. 5 and Table S4). In the three main categories (biological process, cellular component and molecular function) of the GO classification, 26, 15, and 12 functional groups were identified, respectively. Among these groups, the terms cellular process, cell & cell part and binding were dominant in each of the three main categories, respectively. We also noticed a high percentage of genes from categories of biological regulation, metabolic process, multicellular organismal process, regulation of biological process, response to stimulus, organelle and organelle part. The GO analysis showed that the functions of the identified DEGs were involved in various biological processes.

KEGG is a largely publicly available pathway-related database and is useful in searching for genes involved in metabolic or signal transduction pathways that were significantly enriched [24]. Among those genes with a KEGG pathway annotation, 578 DEGs were identified between anagen and catagen. Notably, a specific enrichment of genes was observed for pathways involved in focal adhesion and ECM-receptor interaction. Between anagen and telogen, 447 DEGs with a KEGG pathway annotation were found, and a specific enrichment of metabolic pathways and cell adhesion molecules was noted. Between catagen and telogen, 106 DEGs with a KEGG pathway annotation were found, and a specific enrichment of tight junction and calcium signaling pathways was noted. This suggests that there are considerable differences between the physiological processes at different stages of cashmere goat hair follicle development and cycling.

### Confirmation of Differential Gene Expression by qPCR

**Figure 6:**
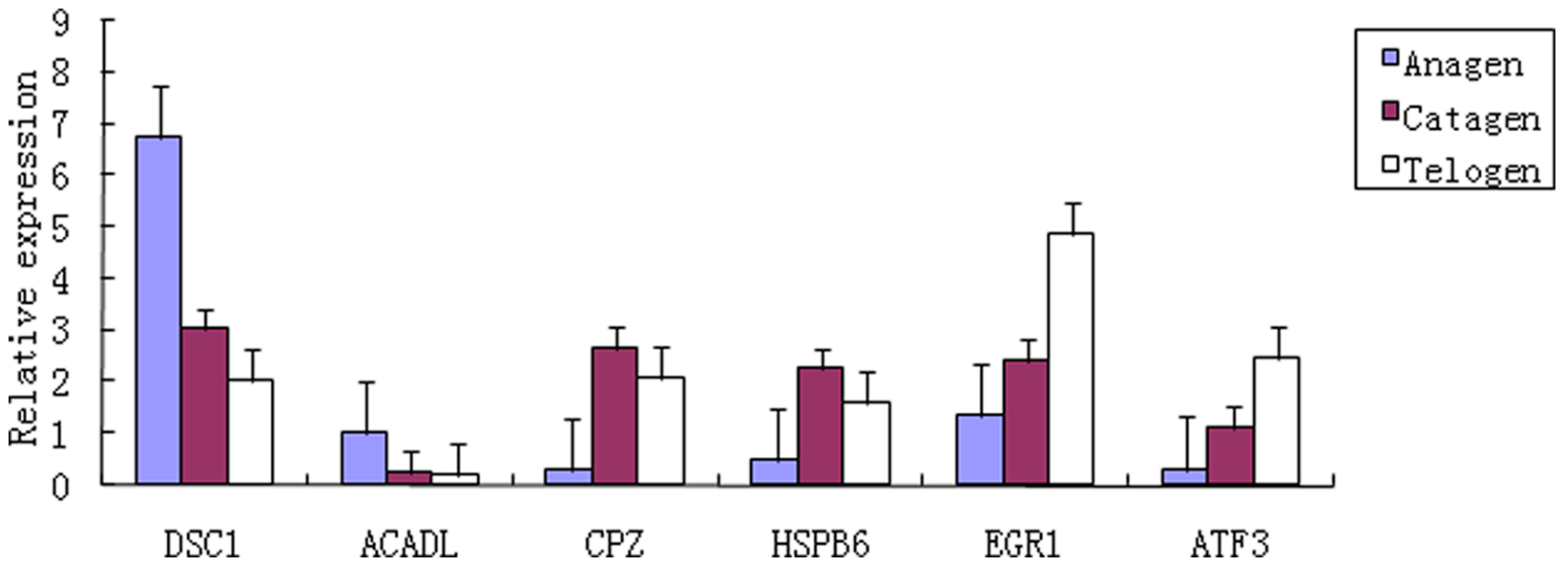
The qPCR validations of DEGs characterised by RNA-Seq. The results verified that these genes were differentially expressed at different hair follicle developmental stages, consistent with the RNA-Seq findings.

We randomly selected six genes to validate the expression profiles obtained by RNA-Seq. The qPCR results verified that these genes were differentially expressed at different hair follicle developmental stages, consistent with the RNA-Seq findings (Fig. 6). Thus, RNA-Seq can provide reliable data for mRNA differential expression analysis from one side as with other similar findings.

## Discussion

Our study offers new information related to gene expression profiles during hair follicle development and cycling in cashmere goats. A limitation of our study is that the entire goat genomic sequence is not available. Thus, our data analysis was based on the ensemble database of the cattle reference genome (Bos_taurus_UMD_3.1/bosTau6). Although the coding sequences between goat and cattle are highly homologous (up to 95% for many genes), using the cattle database to match goat sequences can still be limited. Compared to more than seven million clean reads at each hair follicle development stage in cashmere goats, only about 60% of reads were mapped to the referenced cattle genome and there is still an abundance of unmapped reads.

A comparative expression profiling strategy between different developmental stages was used to identify a subset of genes that were differently expressed. We first discovered how divergent skin hair follicle development is in gene expression between the three stages. Some potential regulators of hair follicle development have been identified. First, the number of DEGs between any two stages shows significant differences, reflecting a cumulative effect on phase traits. The largest difference appears between anagen and catagen, indicating the complexity of the cumulative effect, while the minimum difference occurs between catagen and telogen. Second, expression patterns, GO categories and KEGG pathways associated with DEGs between any two developmental stages show considerable differences. Resemblances of phase differences are observed between anagen vs. catagen and anagen vs. telogen. The phase difference between catagen and telogen is lower. Lastly, the phase difference between catagen and telogen is very different from the other, stages, especially the functional categories of antioxidant activity, channel regulator activity and some GO terms associated with cell killing, pigmentation, rhythmic process, synapse and synapse parts which are unobserved (Fig. 4). This unique pattern between catagen and telogen may indicate that switches in phase-related events occur at the physiological or molecular level during that period.

The hair follicle is the most prominent miniorgan of the skin and one of the defining features of mammalian species. The key prerequisite for hair follicle development and cycling is the molecular communication between the skin epidermis and the underlying mesenchyme [6]–[8], [25], [26]. These transformations are controlled by changes in the local signaling milieu, based on changes in expression/activity of a constantly growing number of cytokines, hormones, neurotransmitters, and their cognate receptors as well as of transcription factors and enzymes that have become recognised as key mediators of hair follicle cycling.

Induction and morphogenesis of the hair follicle is controlled by complex signaling networks within the skin epithelium and between the epithelium and specialised inductive fibroblasts in its adjacent mesenchyme. Many signaling pathways are involved in the hair follicle cycle, such as the Wnt, Shh, TGF-β, EDA/EDAR and BMP pathways. Among these signaling networks, at least three major developmental signaling pathways (Wnt, Shh, and NF-κB/EDAR) are indispensable for hair follicle development and maintenance [27]. In the hair follicle, the EDA/EDAR pathway is required at the earliest stages of hair follicle and epidermal appendage development [28]–[30]. In a similar manner, the Wnt/β-catenin pathway initiates morphogenesis and the onset of hair cycling [31]–[34]. The Shh signaling pathway drives hair progenitor proliferation that is required to build a growing hair follicle [35]–[37]. A recent report showed that Wnt/β-catenin signaling lies both upstream and downstream of the EDA/EDAR/NF-κB pathway [30]. This reveals a complex interplay and interdependence of Wnt/β-catenin and EDA/EDAR/NF-κB signaling pathways in the initiation and maintenance of primary hair follicle placodes. However, the exact relationship between these signaling networks is not fully understood. The current molecular understanding of hair follicle development and cycling relies mainly on mice and human models. Hence, we cannot help but ask whether we were able to identify differentially expressed genes or pathways in cashmere goat hair follicle development and cycling that were previously known to affect hair follicle development. In our KEGG pathway-based analysis, several signaling pathways involved in hair follicle development were also founded including the Wnt, Shh, TGF-β and Notch signaling pathways (Table 3). These results, together with previous findings on a possible involvement in hair follicle development, suggest that these annotated DEGs (Table 3) may play important roles in the signaling pathways between different hair follicle developmental stages of cashmere goats. Indeed, many of the detected differentially expressed genes do not have known roles in hair follicle development. The identification of such novel candidate genes would necessitate further investigation.

**Table 3:** List of possible signaling pathways and genes involved in cashmere goat hair follicle development and cycling.

Signaling pathway	Differentially expressed genes (GeneID)
Wnt	MAP3K7 (NM_001081595), APC (NM_001075986), TBL1XR1 (NM_001193029)
	SFRP4 (NM_001075764), WNT2 (NM_001013001), NFATC4 (NM_001102536)
	JUN (NM_001077827), DYSF (NM_001102490)
Shh	SMO (NM_001192220), WNT2 (NM_001013001)
TGF-β	RPS6KB1 (NM_205816), THBS3 (NM_001101839), ZFYVE21 (NM_001079587)
	EFEMP2 (NM_001076049), THBS2 (NM_176872), FBLN1 (NM_001098029)
	ID3 (NM_001014950), ZFYVE16 (NM_001206200), ID1 (NM_001097568)
	INHBA (NM_174363), THBS4 (NM_001034728)
Notch	DTX3 (NM_001105393), DTX1 (NM_001205734), EGFL7 (NM_001078038)
	DLL4 (NM_001075779)

Various reports have provided evidence that some cytokines and growth factors are involved in hair growth and morphogenesis [38]–[43]. For example, hair follicle growth was suppressed by the external addition of INF-γ, FGF-5 and TGF-β, while such growth was stimulated by KGF, IGF-I and HGF. In this study, our results showed that many cytokines and growth factors were differentially expressed between stages but only a few members (Table 4) were identified as DEGs according to the selection criteria. It mainly included the tumor necrosis factor family, chemokine family, transforming growth factor, insulin-like growth factor, and retinoids. HGF and MSP are all members of the HGF family of cytokines. HGF has been identified as a powerful modulator of hair growth and may play an important role in hair follicle development and cycle [44]. MSP was investigated for its respective action on hair follicles and may be involved in modulating hair growth [45]. Although HGF and MSP were not regarded as DEGs, they had similar expression patterns of highly expressed at anagen and catagen, with relatively low expression at telogen. These expression patterns suggested that HGF and MSP may regulate the hair follicle cycle and maintain hair growth to some extent. A recent review summarised the role of endogenous retinoids in the hair follicle and revealed that retinoids were critically important in the development and maintenance of the hair follicle [46]. Four retinoids, including LRAT, RDH16, SDR9C7 and SDR16C6, were differentially expressed among different stages (Table 4). LRAT, RDH16 and SDR9C7 were reported to be associated with cellular retinol metabolism in the skin [46]–[48]. They may play important roles in different developmental events during cashmere goat hair follicle cycling.

**Table 4:** List of possible cytokines and growth factors involved in cashmere goat hair follicle development and cycling.

Stage	Differentially expressed cytokines and growth factors (GeneID)
Anagen vs.Catagen	TNFSF12 (NM_001205143), CCL21 (NM_001038076), CCL14 (NM_001046585)
	INHBA (NM_174363), TGFB1I1 (NM_001035313), IGF2 (NM_174087)
	RDH16 (NM_001075796), SDR16C6 (NM_001099707), LRAT (NM_177503)
Anagen vs.Telogen	CCL22 (NM_001099162), CXCL3 (NM_001046513), TNFSF12 (NM_001205143)
	LOC504773 (NM_001034220), CCL26 (NM_001205635), CCL21 (NM_001038076)
	CCL14 (NM_001046585), IGF2 (NM_174087), RDH16 (NM_001075796)
	SDR9C7 (NM_001083737)
Catagen vs.Telogen	CCL26 (NM_001205635)

Adenosine is a nucleoside that is naturally present in cells and is known to have various physiological functions, that are mediated via the up- or down-regulation of cAMP, IP3, and IP3/diacylglycerol through the G protein-coupled receptors of the four androgen receptor subtypes ADORA1, ADORA2A, ADORA2B, and ADORA3 [49]. Although the potential effects of adenosine on hair growth have not been fully elucidated, it has been reported to be a potent regulator of hair growth. An adenosine-mediated signaling pathway contributes to minoxidil-induced hair growth [50], and adenosine significantly increased the anagen hair growth rate and the thick hair rate [51]. In particular, adenosine stimulates the growth of hair follicles by triggering the expression of growth factors and β-catenin, and by inducing their downstream target signaling pathways [52]. Adenosine also stimulates fibroblast growth factor-7 gene expression via adenosine A2B receptor signaling in dermal papilla cells [53]. In this study, the ADORA2A gene did not show expression at any stage, while ADORA3 was only expressed during anagen and telogen, although both ADORA1 and ADORA2B genes were normally expressed during each stage. The ADORA1 and ADORA2B genes had similar expression patterns with the trend of high expression during anagen and catagen and then decreased during telogen. Therefore, whether the expression patterns of these receptor genes are related to developmental events of the hair follicle is still unknown. Further investigations will be needed to confirm the roles of adenosine in cashmere goat hair follicle development and cycling.

## Conclusions

Although the genes controlling hair follicle development and cycling in cashmere goats remain largely unknown, the transcriptome analysis presented in this study provides valuable information towards enhancing our understanding of this complex mechanism. The identified differentially expressed genes and pathways could facilitate further investigations of the detailed molecular mechanisms and provide a foundation for future studies of the hair follicle development and cycling in cashmere goats.

## Supporting Information

Table S1 **Differentially expressed genes between anagen and catagen.**
(XLS)

Table S2 **Differentially expressed genes between anagen and telogen.**
(XLS)

Table S3 **Differentially expressed genes between catagen and telogen.**
(XLS)

Table S4 **A list of GO categories for DEGs between different developmental stages.**
(XLS)
